# Vortices enable the complex aerobatics of peregrine falcons

**DOI:** 10.1038/s42003-018-0029-3

**Published:** 2018-04-05

**Authors:** Erwin R. Gowree, Chetan Jagadeesh, Edward Talboys, Christian Lagemann, Christoph Brücker

**Affiliations:** Department of Mechanical Engineering and Aeronautics, City, University of London, London, EC1V 0HB UK

## Abstract

The peregrine falcon (*Falco peregrinus*) is known for its extremely high speeds during hunting dives or stoop. Here we demonstrate that the superior manoeuvrability of peregrine falcons during stoop is attributed to vortex-dominated flow promoted by their morphology, in the M-shape configuration adopted towards the end of dive. Both experiments and simulations on life-size models, derived from field observations, revealed the presence of vortices emanating from the frontal and dorsal region due to a strong spanwise flow promoted by the forward sweep of the radiale. These vortices enhance mixing for flow reattachment towards the tail. The stronger wing and tail vortices provide extra aerodynamic forces through vortex-induced lift for pitch and roll control. A vortex pair with a sense of rotation opposite to that from conventional planar wings interacts with the main wings vortex to reduce induced drag, which would otherwise decelerate the bird significantly during pull-out. These findings could help in improving aircraft performance and wing suits for human flights.

## Abstract (web summary)

Erwin Gowree et al. use wind tunnel experiments with life-sized models of the Peregrine falcon (*Falco peregrinus*) to investigate the aerodynamic basis for the bird’s complex aerobatics. They found that a vortex dominated flow enables the bird to perform high-speed maneouvres with minimal drag.

## Introduction

During stoop, peregine falcon (*Falco peregrinus*), can dive at 39 ms^−1^^1^ to 51 ms^−1^^2^, making it the world’s fastest animal. Diving from high altitude is necessary to build-up such speeds. While soaring, the falcon first climbs with the wings completely stretched out to increase lift, collected from vertical columns of rising air known as ‘thermals’^3^. Within the initial phase of the stoop it adopts a ‘teardrop’ shape (T-shape) where the wings are folded and feathers tucked in a streamlined shape, which is intuitively the lowest drag configuration. The success of the attack largely depends on the manoeuvrability during the second phase of the stoop, when the bird^1^ starts to pull out from the dive, while undergoing two important morphological transformations, namely the cupped-wing shape (C-shape, detail presented in ref. ^4^) and the M-shape (the focus of this manuscript). In C-shape the arms are slighly untucked, creating a cavity between the body and the primary feathers, which are oriented vertically. During the M-shape the arm opens up further into the horizontal plane and the primary feathers are aligned with the axis of the bird to form an M-shaped planform when viewed from the top. This observation was confirmed from the live recordings also reported in ref. ^5^ and is in agreement with previously broadcasted live recording^6–8^. Despite the rapid deceleration in this configuration, the bird is still flying at moderately high speed, which prevents it from stalling and also allows it to spiral back for another attack if needed. This sudden alteration of morphology to achieve such a complex manoeuvre is enabled by the robust musculo-skeletal structure and the superior mechanical strength of the feathers^9^.

In the M-shape, lift increases dramatically and can even reach ~ 18 times its weight^10^, but this theoretically derived figure should be regarded with some scepticism. From a flight mechanics point of view, in order to perform such manoeuvres the bird needs to generate drastic forces during these ‘strenuous’ conditions. Our wind tunnel experiment sheds light on the flow mechanisms that assist the bird in executing this manoeuvre. First, oil flow visualisation technique was used to capture the flow topology and analysis of the near-surface streamlines revealed the presence of strong transverse velocity component and a vortex-dominated flow over the bird. Digital particle image velocimetry^11^ (DPIV) was employed to gather more detail about the development of the vortical structures in the wake of the bird, which interact to reduce the downwash effect. In this technique, the motion of micrometre-sized particles seeded in the flow are traced while illuminated by a laser sheet. Their trajectories are recorded by a high-speed digital camera, and using image-processing algorithm the velocity vector is determined by resolving the displacement of the tracer particle for a known time interval. Further postprocessing of the velocity field helps in resolving the vortices and investigate their dynamics. Complementary Computational Fluid Dynamics (CFD) simulations helped in confirming the experimental findings and provided more details of the flow field in locations where measurements were not possible.

## Results

### Live recordings and model design

**Fig. 1:**
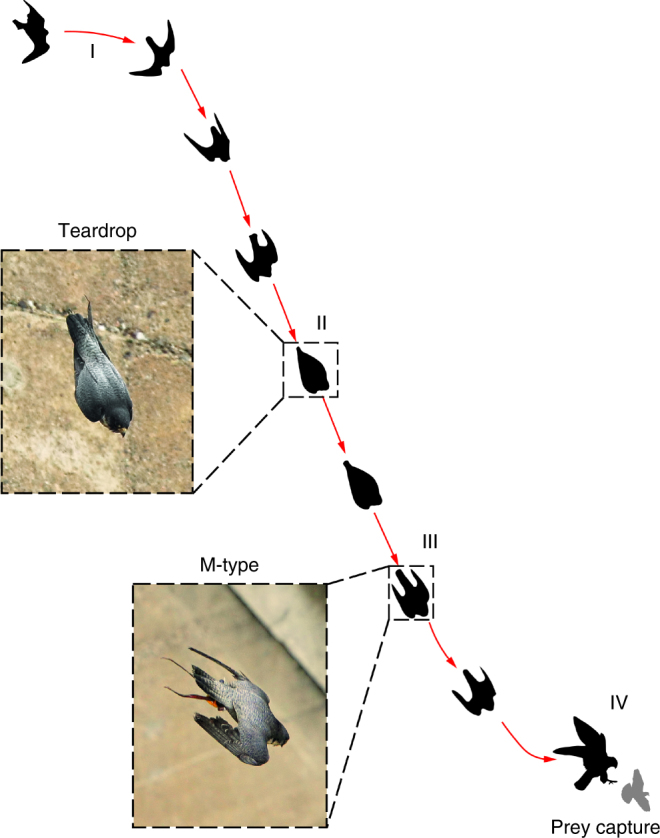
Montage of the flight path of a peregrine falcon in stoop with the corresponding live images while in the Teardrop-shape and the M-shape

From the live recordings during the field experiment conducted at the Oleftal dam in Hellenthal, Germany, the dive path of a trained falcon was reconstructed and this is shown schematically in Fig. 1 together with the silhouette of the wing configurations adopted during various stages of the stoop. The two live images show the real morphology corresponding to the two main silhouettes in the schematic representation. In Fig. 1, phase ‘I’ shows the beginning of the stoop and ‘II’ is when the bird is diving at maximum speed in a T-shape configuration expanded in the corresponding live image. Stage ‘III’ is when the wings are slightly deployed into the M-shape configuration, also shown in the live image. This is usually adopted towards the later phase of the stoop, which is followed by the pull-out manoeuvre in phase ‘IV’ where the bird starts to climb again, while flapping the wings. There is an intermediate stage between ‘II’ and ‘III’ where the wings open up slightly towards the lateral direction with the primary feathers aligned along the vertical plane. This is usually referred as the ‘cupped-wing’ configuration (C-shape) and unlike in the T-shape the the radiale is pointing forward when seen from a planform view. From the field observations, two life-size models were manufactured using rapid prototyping technique and tested in a low-speed wind tunnel. Details of the model design and manufacturing, and the wind tunnel test can be found in the Method section. During the field experiment, upon attaining maximum speed the bird stopped to accelerate and all the forces, mainly aerodynamic and gravitational, are under equilibrium. From the force balance measurement shown in Fig. 13 in Ponitz et al., the equilibrium condition was achieved at an angle of incidence of 5°. This angle was also confirmed during the field experiment, where the bird was seen to maintain this attitude during the dive, until the beginning of the pull-out stage where the angle of attack increases drastically.

### Stoop is characterised by vortex-dominated flow

**Fig. 2:**
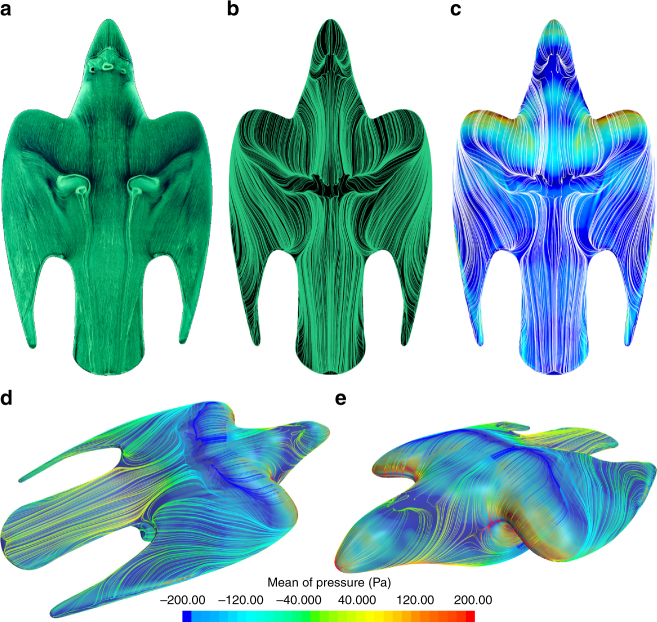
Near-surface streamline pattern to show the topology of the flow over the Falcon. **a** Top view of oil flow visualisation from the wind tunnel experiment. For further details we recommend watching the live video recording, which can be obtained by following the link in the Data Availability section. **b** Top view of the streamlines from the CFD simulation. **c** Top view of the streamlines in white and colour contours of the magnitude of the surface shear stress (red: high shear, blue: low shear). **d**, **e** are isometric views, which show the mean pressure contours lines to show regions of flow acceleration and decelerations due to the complex 3D curvature, overlaid on top, the skin friction contours to correlate regions of flow separation and reattachment

The near-surface streamlines pattern from the oil flow (Fig. 2a) and CFD (Fig. 2b) shows remarkably good agreement, providing confidence in using the CFD for further quantitative analysis of pressure and surface shear stress, *τ*_w_, contours. When analysing the near-surface streamline toplogy using critical point theory^12–16^ the nodes, saddles and foci help in identifying regions of flow separation, re-attachment and vortex generation. Regions of inflections in the streamline usually indicates flow seperation or attachment and the ‘spiralling’ streamline will indicate the presence of a focus, which leads to the formation of a vortex. The streamlined nature of the bird’s wings would lead one to believe that the lift generation process is similar to conventional aerofoil optimised for low drag, hence enabling high-speed performance. However, the highly inflectional streamline patterns in Fig. 2 both experimentally and numerically confirms that the flow over the bird is dominated by vortical structures. Lift generation through unconventional aerodynamics is common in nature where often vortical structures have an important role, mainly for the flight of insects and birds^17–22^. These unsteady flow structures are typically three-dimensional (3D) and is a result of either the motion pattern of the wings, their flexing and bending, or due to their complex morphology during flight. As such, their influence on the lift cannot be explained by classical aerodynamics for planar wings using lifting line theory at steady flight conditions.

Immediately downstream of the neck, in the dorsal region the streamlines first converge and then diverge again, indicating that the streamwise flow accelerates and then decelerates after the neck. The rapid reduction in *τ*_w_ in Fig. 2c indicates the onset of flow separation, which is also confirmed by the detachment node. On the wing, the flow accelerates initially and then the streamlines start to rapidly curl inboard, at angles larger than ± 45° showing strong increase in the magnitude of crossflow velocity component. This strong spanwise flow (~ 8–15% of freestream) feeds the separation bubble and re-energises the boundary layer. A scenario similar to that of two (opposite) jets blowing fluid at an angle with respect to the streamwise flow into the dorsal boundary layer to promote re-attachment of the flow. A flow separation control technique was first demonstrated by Prandtl^23^, where entrainment of higher streamwise momentum fluid is enhanced and, in this case, amplified by the presence of two such jets. The induced crossflow is due to the highly curved 3D surface imposed by the musculo-skeletal structure, which creates a forward sweeping of the wing, with the radiale pointing upstream to form the leading tip while the primary feathers are aligned backwards parallel to the axis of the body. This forward swept part of the overall wing forces the incoming outer wing flow to move inboard. Immediately after re-attachment, the flow converges again, indicating acceleration in the streamwise direction, through a vena-contracta effect, as the diameter of the streamtube effectively reduces to a minimum. This leads to the flow entering the tail feather region being fully attached with parallel longitudinal streamlines. The attached flow is a major prerequisite to ensure effectiveness of pitch control from the tail, where the feathers are twice stiffer than other avians^9^ and hence providing sufficient resistance to bending and avoid uncontrolled deflection under aerodynamic loads. On aircraft, this issue with tail ineffectiveness is mitigated by mounting it off the axis of the fuselage in a ‘T-tail’ configuration to keep the elevators in clean flow, but the bird applies a natural preconditioning of the upstream flow.

### Horn, horseshoe and dorsal vortices

**Fig. 3:**
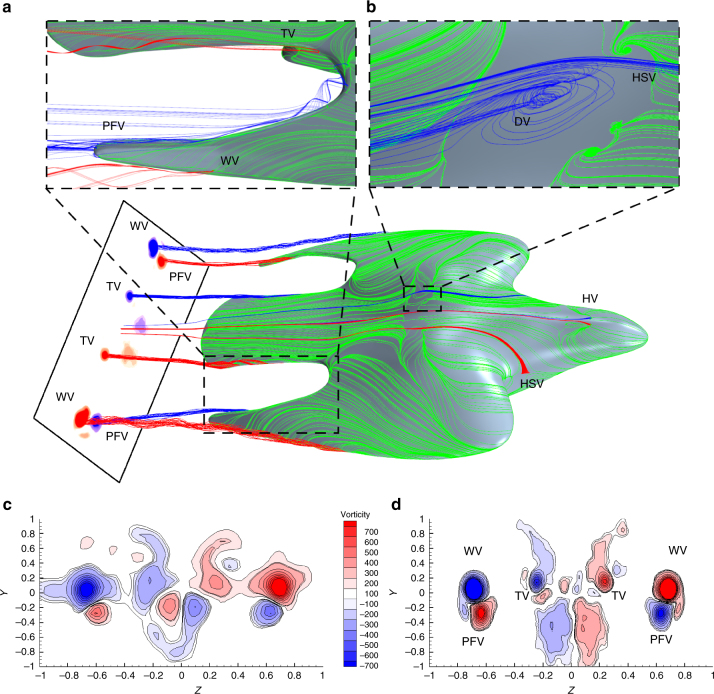
Evolution and passage of the vortical structures over the bird. The wake plane (0.2 body length downstream of the tail of the bird) shows the contours of the streamwise vorticity magnitude (colour based on the sense of rotation, red: counter-clockwise, blue: clockwise). The path of these vortices can be tracked by following each of the corresponding streamlines. Origin of the vortices is indicated in enlarged sub-figures. **a** Origin of the wing vortex (WV) and tail vortex (TV), the red streamline represents the wing vortex and the blue the primary feather vortex (PFV). **b** Origin of the dorsal vortex (DV) and the interaction with the horseshoe vortex (HSV). **c** DPIV measurement in the wake plane compared with the results from the CFD simulation **d** in the same plane

Starting from the front of the bird, Fig. 2 indicates the presence of two detachment nodes and two foci, which confirms the formation of a pair of vortices similar to the ‘Werlé-Legendre’ or ‘horn’ vortices present on slender cylindrical bodies at angles of attack^15, 16^. Here, the horn vortices are due to the blunt forehead of the bird facing the flow at an incidence. The saddle point in the pressure contour lines, ahead of the valley between the wing and neck junction in Fig. 2 also shows the presence of a horseshoe vortex on each side of the bird, which wraps around the inboard region of the wing. The footprint of the horseshoe vortex on the surface is also seen in the oil flow visualisation in Fig. 2a and its origin is depicted in the centre of Fig. 3. In Fig. 3b, the foci in the dorsal separation region show the emergence of a pair of dorsal vortices, which interact with the horseshoe vortex while they propagate downstream. The CFD simulation confirmed that the horn, horseshoe and dorsal vortices are relatively small, and therefore their influence is localised. However, they are all of the same sense of rotation on each side of the body and interact with each other in the dorsal region, again re-energizing the boundary layer over the tail similar to vane vortex generators^24^.

### Formation of a primary feather vortex

Other regions of highly curved streamlines are seen in the outboard region of the wing in Fig. 2. This indicates the presence of a significantly strong wing vortex shown in detail in Fig. 3a where it is labelled as ‘WV’. Interestingly, the channelling of the flow in the gap between the tail and the primary feathers promotes the formation of other unexpected vortices at the wing-tip and the wing-tail juncture (red streamline), shown in Fig. 3a. These vortices merge to form a primary feather vortex (PFV), which rotates in the opposite sense to the wing vortex. This gap also promotes the formation of a fairly strong vortex in the outboard region of the tail. The presence of a vortical structures over the tails of avians has been reported previously^25^; however, the absence of the forebody in that particular experiment raises a few concerns about how representative the model was with respect to real birds where the upstream and downstream flow are tightly coupled. In this case, the wing vortices, PFV and tail vortices are significantly stronger than the ‘horn’, horseshoe and dorsal vortices as seen from the vorticity magnitude contours in the wake plane in Fig. 3c and d. Owing to 3D surface curvature of the bird model, DPIV measurement directly over it proved to be extremely challenging, therefore the measurement plane was located in the wake downstream. From Fig. 3c, d, the dominant vortices have been resolved by both the DPIV measurements and the CFD simulation, respectively. The main difference between the experiment and the CFD lies in the middle region where contribution from the bird’s body wake is slightly more pronounced in Fig. 3d. This mismatch is due to the sting mount in the experiments, which was not considered during the meshing for the CFD simulations. The junction between the bird’s body and the mount promotes the formation of a horseshoe vortex, which wraps around the mount, is common for surface-mounted cylinder^16^.

## Discussion

**Fig. 4:**
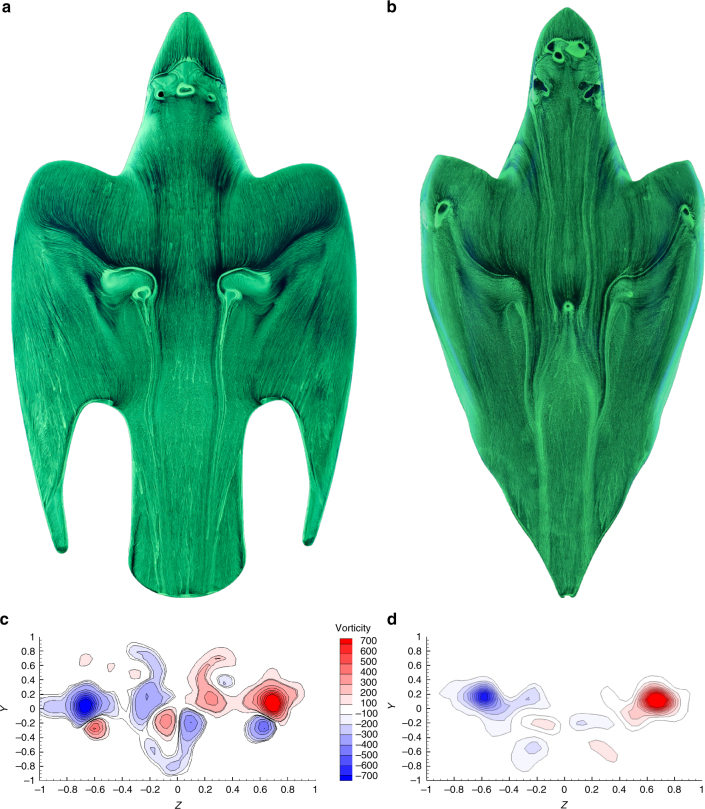
A comparison between the M-shape and the C-shape. **a**, **b** The oil flow visualisation over the M-shape and the C-shape, respectively, where the spanwise flow in the dorsal region appears to be more significant on the M-shape. **c**, **d** The DPIV measurement in the wake downstream of the M-shape and the C-shape models, respectively

Here we have shown that considerably strong spanwise flow exist on the wings, which more importantly forces the flow from the outer region of the wing inboard. This flow is a result of the forward swept effect of the wings with the radiale as the upstream pointing tip and has so far not been reported on other birds, e.g., in the case of swifts^22^. Further validation of the enhanced manoeuvrability achieved from the M-shape is obtained while comparing it against the C-shape. Figure 4b shows that here the spanwise flow is very weak, this result in a small region of separated flow in the dorsal region and over the tail. This could be a hinderance if the bird wants to pitch slightly, so as to re-establish equilibirum or modify the dive path. Hence, a possible reason to why the bird is often seen to open up its primaries and tail momentarily also during the high-speed dive, thereby allowing it to trim its attitude along an efficient dive path and again confirming the importance of the M-shape for manoeuvring. Hence, the M-shape morphology can be considered as a wing with a forward swept leading edge, which directs the spanwise flow in-board to promote flow reattachment and a highly cranked outboard region generating a raked wing-tip, known to reduce induced drag^26, 27^. For planar wings, an increase in lift leads to increase of the induced drag^23^ and in theory this effect would cause the bird to lose speed considerably. However, the counter-rotating PFVs reduce the downwash and hence ensures that the induced drag is not greatly incremented. The absence of the counter-rotating PFVs in the C-shape in Fig. 4d shows further evidence of this mechanism of induced drag reduction.

The tail vortices generate additional loading on the tail feathers which upon deflecting upward or downward generates pitching moment, whereas twisting generates rolling moment. Again, the falcon’s tail is able to act as an empenage due to its superior mechanical property. Together with the additional lift from the preconditioned, attached flow the pitching moment generated by tail is greatly increased, thus enhancing manoeuvrability. Furthermore, the suction effect induced by the strong wing vortices also contribute to the pitching moment, which can be tuned by extending the wing forward or backward, hence shifting the mean aerodynamic centre relative to the centre of gravity of the bird. As mentioned above, the flow pre-conditioning plays an important role in ensuring that the tail sees an attached, almost parallel flow. In addition, by changing the local angle of attack of the primaries the bird can roll and coupled with the twisting of the tail about the longitudinal axis, yaw control could be established.

From a flight mechanics point of view, the manoeuvrability of falcons is greatly enhanced by the vortical structures, which control the flow by suppression of separation and also due to additional lift generated by suction from stronger vortices similar to that on delta-wings for flight control purposes. Keeping the M-shape during the flight requires a robust musculo-skeletal structure, specially to prevent lateral movement of the wrists and ensuring that the wings are adducted close to the body. As the feathers of *F. peregrinus* are proven to be adapted to these high loads, we speculate that the same must hold for the muscles, bones and the joint between the humerus and forearm, as they have special importance for the stabilisation of the body form during a dive.

## Methods

### Bird model

State-of-the-art high-speed optical tracking equipments employed during the field experiment helped in resolving the flight path and wing configurations at various intervals during the stoop^5^. The geometry of the bird was obtained by a 3D scan of a stuffed peregrine falcon with its wings fixed accordingly with the morphology observed from detailed analysis of the recordings during the field experiment. These coordinates were imported into a CAD package for further fine tuning of the surface contours required to manufacture the life-size wind tunnel models using rapid prototyping (3D printing) and for the CFD simulation. The surface of the 3D printed model was polished manually until it was deemed smooth, similar to the surface texture of the feathers of a peregrine falcons. Owing to the high mechanical strength of the tail and primary feathers reported by ref. ^9^, we assumed that they are acting as aerodynamic surfaces with minimal deflection and hence negligible effect to the overall meanflow due to plumage deformation. The life-size model of length 0.40 m and span 0.22 m was mounted horizontally on a sting fixed between the anal region of the bird and the floor of the test section (0.8 m high, 1.12 m wide and 1.8 m long) of the low-speed wind tunnel facility of the Handley Page Aeronautics Laboratory at City, University of London. It was set at an angle of incidence of 5° and the experiment was conducted at a freestream velocity of 22.5 m/s following ref. ^5^. This corresponds to a Reynolds number of ~ 5.8 × 10^5^, based on the length of the bird. The angle of attack is defined as the angle between the chord-line (here passing through the body of the bird) and the direction of the freestream flow in the tunnel. In free flight, it is the angle between the chord and the trajectory of the bird. This angle was derived from a combination of field observations (shown schematically in Fig. 1) and wind tunnel testing in Freiberg, led by C. Brücker and reported by Ponitz et al. As we are mainly interested in the flow physics at this relatively low incidence, the blockage effect was assumed to be negligible. Following ref. ^9^, the effect of plumage was assumed to be negligible on the mean flow.

### Flow visualisation technique

Surface flow visualisation technique comprising a mixture of white spirit (Naphtha), DayGlo powder and Oleic acid was employed to capture the topology of the flow over the bird model. The mixture was applied on the surface using a fine bristled paint brush, right before the tunnel was operated. The evolution of the oil film was recorded and the final fully developed pattern was photographed under ultraviolet lighting to increase definition. Owing to shear stress between the surface and the fluid near the surface a pattern of streamlines also known as skin friction lines is generated, indicating the trajectory of the near-surface flow. Topological representation of these near-surface streamlines helped in identifying and interpreting regions of detached flow and the origins of vortices using critical point theory. Analysis of the video recordings of the transient oil-film motion was very helpful in identifying regions of separation and vortex formation, which was difficult to interpret only from the still image of the finally dried pattern.

### Digital particle image velocimetry

A two-camera stereo version of a standard TSI DPIV system is used; illumination is done with a 4 mm-thick double-pulsed Nd:YLF laser (Litron LDY303, 527 nm wavelength, 20 mJ/pulse) expanded to a sheet with a cylindrical lens. Two high-speed cameras (Phantom Miro M310, Vision Research, 1280 × 800 pixels) are mounted on either side of the test section and view the wake of the bird using Scheimpflug tilt-shift mechanism and a macro lens (Tokina 100 mm f/2.8). The fields of view of the two cameras were adjusted such that a common field of view encompassed the entire wake of the bird model, with the centreline of the bird lining up with the centre of the field of view. The cameras viewing angles were approximately 40 degrees each, with a Scheimpflug angle of ~ 6° each. They were calibrated to account for the angular distortion against a two-plane target having calibration marker points that alternate between two depths. Olive oil droplets with an average size of 1 μm generated by a seeding generator (Laskin-nozzle type) were used as seed particles, injected downstream of the bird model. DPIV image pairs were acquired at a rate of 250 Hz for 4 s resulting in a total of 1000 image pairs. The raw image pairs from both the cameras were pre-processed using TSI Insight 4G software and a robust cross-correlation algorithm determined the velocity vector field. The spot size for the first pass of the vector field computation was 64 × 64 pixels, with a 50% overlap grid spacing. The spot size was reduced to 32 × 32 pixels for the second pass, with a maximum displacement allowance of 16 pixels. A Gaussian mask was implemented with a FFT correlator to compute the correlation function. The computed vectors for each camera was then subjected to the perspective spatial calibration to produce the three components of velocity. The vector fields were validated using a local median filter (3 × 3) and any missing vectors were interpolated by using a local mean. Any spurious vectors were discarded and they were replaced by an interpolated vector, as mentioned before. The amount of spurious vectors were less than 2% of the entire vector dataset. The vorticity field shown in Fig. 3c were obtained from the average of the full sample.

### Computational fluid dynamics

All CFD simulations are conducted using the commercial software, STAR CCM+ v.12, which is capable of implicit unsteady Reynolds-Averaged Navier-Stokes (RANS) and Large Eddy Simulation (LES) calculations. The computational domain was decomposed in a global cartesian grid and near-surface prism layers were generated from a surface mesh. The initial mesh was based on an anisotropic hexahedral trimmed grid and has approximately 57 million cells where the minimum grid spacing was defined on the basis of *y*^+^ ≤ 1 to resolve the fine scales of the boundary layer^28^. A second-order accurate scheme was selected for the spatial and temporal discretisation^29^. The initial RANS simulations were regarded as converged if the residuals of the momentums (*X*, *Y*, *Z*) and the energy dropped below *ε* = 10^−5^. To ensure that neither timestep nor mesh induced errors influenced the numerical results from the LES, all simulations were recalculated using a coarser and finer mesh respectively for a smaller and bigger time step. Further detail is presented as Supplementary Material where Supplementary Fig. 1 shows evidence of convergence. As the CAD model is an exact replica of the original falcon model design, the results could be directly compared with the experimental data.

### Data availability

The live recording of the surface oil-flow visualisation, the DPIV and the CFD simulation results are available at ‘figshare’ under 10.6084/m9.figshare.5928229

## Electronic supplementary material

Supplementary Information(PDF 149 kb)

## References

[1] Alerstam T (1987). Radar observations of the stoop of the Peregrine Falcon Falco peregrinus and the Goshawk Accipiter gentilis. Ibis.

[2] Peter D, Kestenholz M (1998). Sturzflüge von wanderfalke Falco peregrinus und wüstenfalke *F. pelegrinoides*. Der Ornithol. Beob..

[3] Pennycuick CJ (1975). Avian Biology: Mechanics of Flight.

[4] Ponitz B, Triep M, Brücker C (2014). Aerodynamics of the cupped wings during peregrine falcon’s diving flight. Open J. Fluid Dyn..

[5] Ponitz B, Schmitz A, Fischer D, Bleckmann H, Brücker C (2014). Diving-flight aerodynamics of a peregrine falcon (*Falco peregrinus*). PLoS. ONE.

[6] National Geographic. High-velocity falcons (2007) https://video.nationalgeographic.com/video/falcon_peregrine_velocity.

[7] National Geographic. World’s deadliest: superfast flyer makes a kill (2013) https://video.nationalgeographic.com/video/worlds-deadliest/deadliest-peregrine-falcon.

[8] BBC. Peregrine falcon sky dive-inside the perfect predator (2010) https://www.youtube.com/watch?v=legzXQlFNjs.

[9] Schmitz A (2015). Morphological properties of the last primaries, the tail feathers, and the alulae of *Accipiter nisus*, *Columba livia*, *Falco peregrinus*, and *Falco tinnunculus*. J. Morphol..

[10] Tucker VA (1998). Gliding flight: speed and acceleration of ideal falcons during diving and pull out. J. Exp. Biol..

[11] Raffel, M., Willert, C. E., Wereley, S. & Kompenhans, J. *Particle Image Velocimetry: A Practical Guide* (Springer, New York, 2007).

[12] Poincaré H (1882). Les points singuliers des equations différentielles. C. R. Acad. Sci. Paris..

[13] Legendre R (1956). Seperation de l’ecoulement lamnaire tridimensionel. Rech. Aeronaut..

[14] Lighthill, J. M. *Laminar Boundary Layers*. (Oxford Univ. Press, London, 1963).

[15] Tobak M, Peake DJ (1982). Topology of three-dimensional separated flows. Annu. Rev. Fluid. Mech..

[16] Délery JM (2001). Robert Legendre and Henri Werlé: toward the elucidation of three-dimensional separation. Annu. Rev. Fluid Mech..

[17] Weis-Fogh T (1973). Quick estimates of flight fitness in hovering animals, including novel mechanisms for lift production. J. Exp. Biol..

[18] Lighthill MJ (1973). On the Weis-Fogh mechanism of lift generation. J. Fluid Mech..

[19] Ellington CP, van den Berg C, Willmott AP, Thomas ALR (1996). Leading-edge vortices in insect flight. Nature.

[20] Birch MJ, Dickinson MH (2001). Spanwise flow and the attachment of the leading-vortex on insect wings. Nature.

[21] Srygley RB, Thomas ALR (2002). Unconvetional lift-generating mechanisms in free-flying butterflies. Nature.

[22] Videler JJ, Stamhuis EJ, Povel GDE (2004). Leading-edge vortex lifts swifts. Science.

[23] Prandtl, L. *Führer Durch die Strömungslehre: Grundlagen und Phänomene*. (Springer, Berlin, 1931)

[24] Lin JC (2002). Review of research on low-profile vortex generators to control boundary-layer separation. Prog. Aerosp. Sci..

[25] Maybury WJ, Rayner JMV, Couldrick LB (2001). Lift generation by the avian tail. Proc. R. Soc. B..

[26] Zahm, A. F., Bear, R. M. & Hill, G. C. Lift and drag effects of wing-tip rake. *NACA-TR-140* (1923).

[27] Kroo I (2001). Drag due to lift: concepts for prediction and reduction. Annu. Rev. Fluid Mech..

[28] Meyers J, Bernhard G, Sagaut P (2008). *Quality and Reliability of Large-Eddy Simulations*.

[29] Peyret, R. *Handbook of Computational Fluid Mechanics*. (Academic Press, London, 2011).

