# Supplementary material for: Vortices enable the complex aerobatics of peregrine falcons
Source: Commun Biol. 2018 Apr 5;1:27. doi: 10.1038/s42003-018-0029-3 (PMC6123743; doi:10.1038/s42003-018-0029-3)
Supplement: Supplementary file 1 — Supplementary Information(PDF 149 kb) [file 42003_2018_29_MOESM1_ESM.pdf]

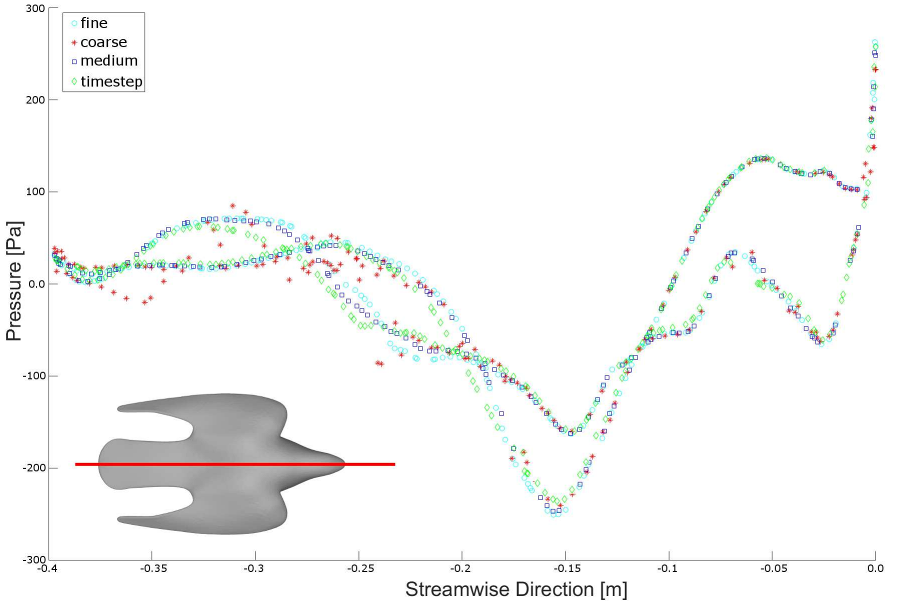

Supplementary Figure 1. shows the pressure distribution along the symmetry plane for different mesh and time-step of the LES evaluation process. 'Medium' represents the initially computed LES using the reference timestep and mesh size. Comparing these results to bigger ('coarse') and smaller ('fine') cell sizes, one can see that a refined mesh barely shows any differences to the reference case. Additionally, the basic timestep of the reference mesh have been changed to study timestep induced errors. In general, a smaller timestep represented by the green diamonds (timestep) yields to similar results compared to the reference case (medium) and consequently timestep and mesh induced errors can be regarded as negligible.
